# Dynamics of subjective pattern of health in frequently ill adolescents

**DOI:** 10.1192/j.eurpsy.2022.583

**Published:** 2022-09-01

**Authors:** I. Shishkova, E. Pervichko

**Affiliations:** 1 Ryazan State Medical University named after I.P. Pavlov, Faculty Of Clinical Psychology, Ryazan, Russian Federation; 2 Lomonosov Moscow State University, Faculty Of Psychology, Moscow, Russian Federation; 3 Pirogov Russian National Research Medical University, Clinical Psychology Department, Moscow, Russian Federation

**Keywords:** frequently ill adolescents, health, subjective pattern of health, attitude toward health in adolescents

## Abstract

**Introduction:**

It is known that frequently ill adolescents are significantly more likely to have a disharmonious subjective pattern of health and associated maladaptive behavior in the field of health.

**Objectives:**

To study dynamics of subjective pattern of health in frequently ill adolescents.

**Methods:**

The sample: 57 frequently ill adolescents (mean age 10.6±0.1) in 2014-2015, 2017 and 2019. We used: The method of unfinished sentences about health (Yakovleva, 2014), “Index of attitude toward health” (Deryabo, Yasvin, 1999).

**Results:**

The index of emotionality has decreased (98.93 vs 84.87, p=0.015), while the cognitive indicator of the attitude toward health remains unchanged. The rate of positive self-assessment of health reduces and the rate of negative self-assessment of health increases. Frequently ill teenagers in older age are more likely to give an objective definition of the disease and less likely to emotionally assess it. At a young age they often pointed out active lifestyle as the main reason for health, with age the psychological characteristics of a person (strength of will, cheerfulness, etc.) this reason becomes. The rate of health recognition as the absolute value of man decreases (in the second cut answers, this category accounted for only 24.4%), but at a later age teenager show “adult” category of “quality of life” (noted by 7.4% of older teenagers).

**Conclusions:**

The study of the dynamics of the subjective pattern of health in frequently ill adolescents has high practical significance due to the possibility of preventive and correctional work with these adolescents. Research is supported by the Russian Science Foundation, project No. 21-18-00624.

**Disclosure:**

Research is supported by the Russian Science Foundation, project No. 21-18-00624.

